# Modeling the effect of heat treatment on fatty acid composition in home-made olive oil preparations

**DOI:** 10.1515/biol-2020-0064

**Published:** 2020-08-24

**Authors:** Dani Dordevic, Ivan Kushkevych, Simona Jancikova, Sanja Cavar Zeljkovic, Michal Zdarsky, Lucia Hodulova

**Affiliations:** Department of Plant Origin Foodstuffs Hygiene and Technology, Faculty of Veterinary Hygiene and Ecology, University of Veterinary and Pharmaceutical Sciences Brno, 61242, Brno, Czech Republic; Department of Technology and Organization of Public Catering, South Ural State University, 454080, Chelyabinsk, Russia; Department of Experimental Biology, Faculty of Science, Masaryk University, Kamenice 753/5, 62500, Brno, Czech Republic; Department of Genetic Resources for Vegetables, Centre of the Region Haná for Biotechnological and Agricultural Research, Medicinal and Special Plants, Crop Research Institute, 78371, Olomouc, Czech Republic; Department of Phytochemistry, Centre of Region Haná for Biotechnological and Agricultural Research, Faculty of Science, Palacky University, 78371, Olomouc, Czech Republic

**Keywords:** virgin olive oil, refined olive oil, saturated fatty acids, monounsaturated fatty acids, polyunsaturated fatty acids, cross-correlation analysis

## Abstract

The aim of this study was to simulate olive oil use and to monitor changes in the profile of fatty acids in home-made preparations using olive oil, which involve repeated heat treatment cycles. The material used in the experiment consisted of extra virgin and refined olive oil samples. Fatty acid profiles of olive oil samples were monitored after each heating cycle (10 min). The outcomes showed that cycles of heat treatment cause significant (*p* < 0.05) differences in the fatty acid profile of olive oil. A similar trend of differences (*p* < 0.05) was found between fatty acid profiles in extra virgin and refined olive oils. As expected, the main differences occurred in monounsaturated fatty acids (MUFAs) and polyunsaturated fatty acids (PUFAs). Cross-correlation analysis also showed differences between the fatty acid profiles. The most prolific changes were observed between the control samples and the heated (at 180°C) samples of refined olive oil in PUFAs, though a heating temperature of 220°C resulted in similar decrease in MUFAs and PUFAs, in both extra virgin and refined olive oil samples. The study showed differences in fatty acid profiles that can occur during the culinary heating of olive oil. Furthermore, the study indicated that culinary heating of extra virgin olive oil produced results similar to those of the refined olive oil heating at a lower temperature below 180°C.

## 1 Introduction

Olive oil, especially extra virgin olive oil, is the major source of fat in the so-called Mediterranean diet that very often has positive health benefits. The reason for this attribute is mainly the lower content of saturated fatty acids (SFAs) and the higher content of polyphenolic compounds. The fatty acid profile is important for the quality and stability of oils. Oleic acid (C18:1) is a monounsaturated fatty acid (MUFA), and it is more stable than polyunsaturated fatty acids (PUFAs). PUFAs are usually present in lower percentages in olive oil [1,2,3,4]. Cold-pressed edible plant oils, such as extra virgin olive oil, are usually added to different salads. This may mean that they are consumed without heat treatment. Recently, these types of edible plant oils have been used for cooking, as part of different culinary techniques and recipes. In this case, even cold-pressed edible plant oils are heated at high temperatures. Such heating processes can affect the fatty acid profile of these oils, for instance, heating increases the trans fatty acids, which have potentially harmful health effects [5].

Different culinary conditions, especially the temperature and the time of heat processing, can influence the fatty acid composition of oils [6,7]. The type of oil also influences the changes in oil and its stability during heat treatment due to difference in fatty acid profiles. SFAs are more stable than unsaturated fatty acids, and similarly MUFAs are more stable when compared to PUFAs [8]. The advantage of olive oil is the higher content of MUFA (oleic acid), and this property gives olive oil a higher level of oxidative stability than that of other edible plant oils with a higher content of PUFAs. Cold-pressed olive oil or extra virgin olive oil has the additional advantage of high levels of polyphenols, tocopherols, and carotenoids [4,9,10]. The fatty acid profile of olive oil is influenced by olive variety, weather, environment, and harvest conditions, as well as agronomic and technologic factors [11,12].

Olive oil, especially extra virgin olive oil (cold-pressed olive oil), is considered beneficial for the health of consumers due to the presence of phenolic compounds, such as hydroxytyrosol (which improves radical stability) and oleuropein. Such compounds are involved in inhibiting inflammatory processes. They additionally prevent liver damage by reducing oxidative stress, mitochondrial dysfunction, endoplasmic reticulum stress, and insulin resistance [13,14].

Deep frying is a frequently used culinary practice. The temperature during deep frying is usually around 180°C. This practice is popular among consumers since deep fried food usually have attractive sensory properties [15,16]. The legislation concerning the fatty acid profile of different olive oils is given in percentages, with a key emphasis on oleic fatty acid (C18:1) [17].

This study aimed to simulate home-based heat treatment of olive oil and to measure the changes in the profile of fatty acids due to repeated heating cycles.

## 2 Materials and methods

### 2.1 Heat treatment of olive oil samples

The samples (*n* = 10) used in the experiment consisted of refined olive oils (*n* = 4) and multivariate extra virgin olive oils (*n* = 6; olive oil purchased in retail markets in the Czech Republic, originating from Spain and Greece). Each purchased olive oil had around 18 months for the expiration date. The purchased olive oil samples were packed in glass bottles (volume: from 0.5 to 1 L). The labeling of the olive oil samples indicated that they had peroxide value (PV) and free fatty acid (FFA) content below the allowed limits proposed by Codex Alimentarius (refined olive oil: PV < 5 mEq of active oxygen/kg oil, FFAs < 0.3 mg KOH/g oil; cold-pressed olive oil: PV < 20 mEq of active oxygen/kg oil, FFAs < 0.8 mg KOH/g oil; the standard limits were written on the labeling of extra virgin olive oil samples). Part of the olive oil sample was taken to serve as a control sample without heat treatment. The remainder of the olive oil sample (300 mL) was heated in open glass tubes (250 mL glass tubes used for Kjeltec 2300 digestion) at 180°C and 220°C, in an oven (Professional Ovens GARB-IN, Model:23 GM). The heat treatment lasted for 10 min and was repeated for three cycles (3 × 10 min). Between each cycle, part of the olive oil sample (100 mL) was taken for analysis (of fatty acid composition) and the remainder was heated again. Between heat cycles, the samples were allowed to cool for 20 min.

### 2.2 Fatty acid composition

**Figure 1:**
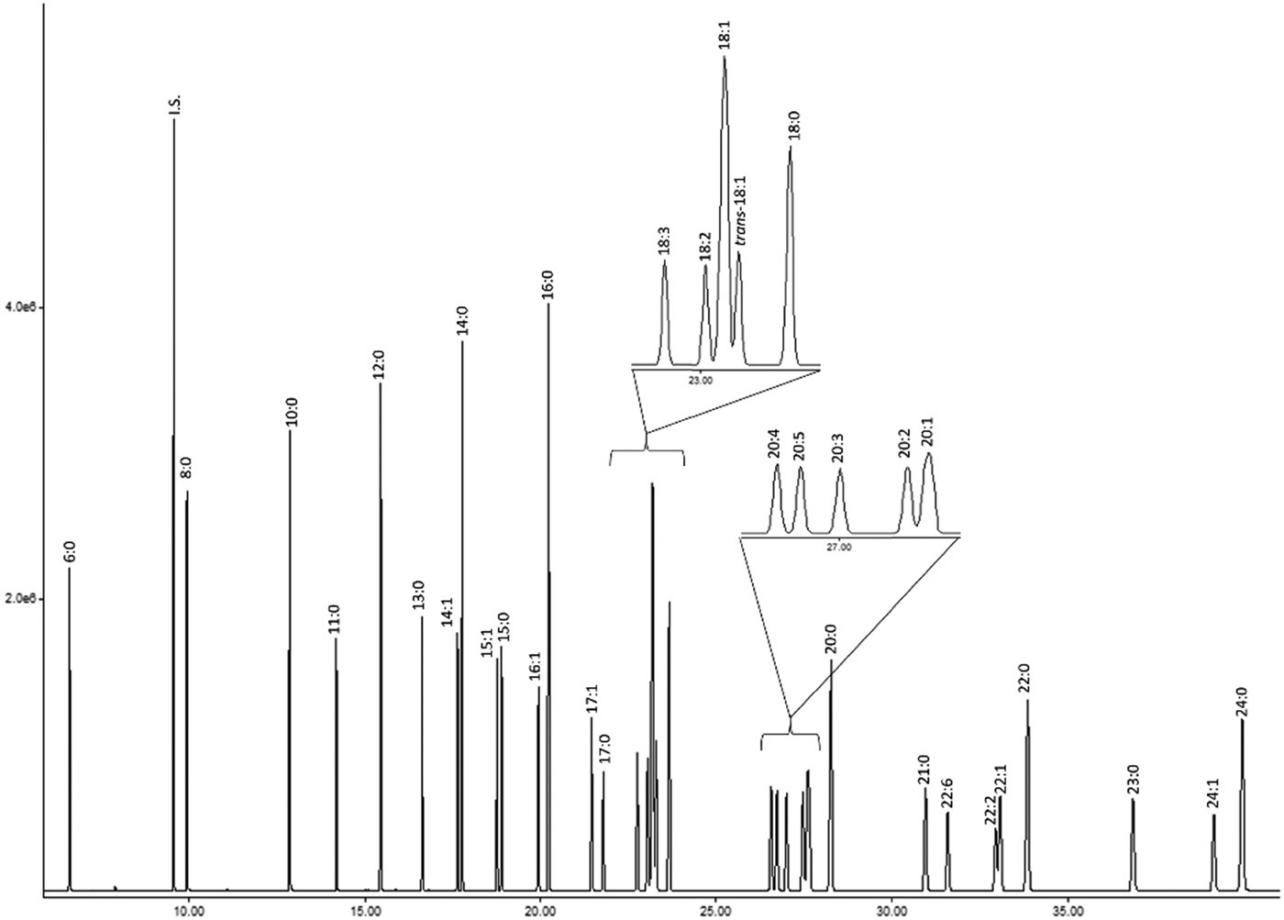
Chromatogram of FAME standards (Supelco).

Fatty acids were methylated with 0.5 M NaOMe/MeOH solution and extracted into *n*-hexane. The resulting fatty acid methyl esters (FAMEs) were analyzed by the gas chromatography–mass spectrometry method on the Agilent system (GC 7890 A; MSD 5975C series II) on a fused silica HP-5MS UI column (30 m × 0.25 mm × 0.25 mm) and carrier gas He (1.1 mL/min). The temperature was programmed at 40°C for 2 min, 10°C/min to 200°C, and finally 2°C/min to 250°C for 2 min. Post run of 15 min was set at 310°C. The temperature of the injection port was 250°C and that of the detector was 280°C. Ionization was performed in the EI mode (70 eV). Identification was performed by comparison of retention times and mass spectra with authentic standards (Supelco. Merck KGaA, Darmstadt, Germany), and the results were presented as mg/mL. The results were calculated according to the official method for fatty acid profile determination [18,19,20]. The successful separation of the evaluated FAMEs, including *n*-undecane that was used as an internal standard, is shown in Figure 1.

### 2.3 Antioxidant profile

Chlorophyll, carotenoid, and polyphenol contents were measured in the samples of olive oil before (control samples) and after three heating cycles at 180°C and 220°C. All the samples were measured in triplicates to reduce the possibility of error.

Carotenoid, polyphenol, and chlorophyll contents were evaluated on a Cecil Instrument spectrophotometer (CE7210). The wavelength for calculating carotenoids was 445 nm. The cuvettes were rinsed with *n*-hexane to avoid mixing of samples. The carotenoid contents were expressed as β-carotene. The quantification was done according to the extinction coefficient and the results were expressed as mg/kg [21].

The polyphenol contents were determined by a standard method using the Folin–Ciocalteu reagent at a reading absorbance of 765 nm. The procedure included the use of the Folin–Ciocalteu reagent previously diluted with distilled water (1:10) and sodium carbonate. The incubation of the sample (1 g), mentioned chemicals, and distilled water was done in the dark and lasted for 30 min. Results were expressed as gallic acid equivalents (mg/kg) [22].

Chlorophyll contents were measured and calculated at the wavelengths of 630, 670, and 710 nm. The samples were not diluted, and the cuvettes were rinsed with *n*-hexane. Results were expressed as pheophytin (mg/kg) [23].

### 2.4 Statistical analysis

The results were calculated (mean ± SD) in the program Microsoft Office Excel 2007 (Microsoft Corp., Redmond, WA, USA). Triplicate sampling was used in all data calculations. Statistical significance (*p* < 0.05) was estimated by the analysis of variance. The Shapiro–Wilk test was used to determine the results’ normality, whereas Levene’s test was used to estimate the homogeneity of variances. The results with normal distribution, homogenous variances, and significant differences between independent variances were found by Tukey’s *post hoc* test. The Games–Howell test was used for the results without normal distribution and unequal variances. Correlations between heating cycles and fatty acid profile changes were measured by Pearson’s correlation analysis. The overall differences between independent variances were checked by principal component analysis (PCA). SPSS 20 statistical software (IBM Corporation, Armonk, USA) was used for the mentioned statistical analysis.

The correlation analysis was carried out to study the temporal correlations between the control, first heating cycle, second heating cycle, and third heating cycle for both temperature regimes of 180°C and 220°C for extra virgin and refined olive oil samples. The values of the defined function parameters (namely, SFAs, MUFAs, and PUFAs) were used in the correlation study. Cross-correlation plots were built by software package Origin7, based on the cross-correlation results obtained using SPSS 20 statistical software (IBM Corporation, Armonk, USA).

## 3 Results and discussion

Fatty acid profile changes, during the three heating cycles, in extra virgin and refined olive oil samples are shown in Tables 1–4 and Tables S1–S4. Statistical significant (*p* < 0.05) differences were not found in concentrations of the following SFAs: caproic (C6:0), caprylic (C8:0), capric (C10:0), lauric (C12:0), palmitic (C16:0), stearic (C18:0), heneicosanoic (C21:0), tricosanoic (C23:0), and lignoceric (C24:0) fatty acids in the samples of extra virgin olive oil heated in three cycles at 180°C. Although caproic acid was under detectable limits after three cycles of heating, myristic acid (C14:0) increased (*p* < 0.05) after three cycles of heating. The same trend occurred with pentadecylic acid (C15:0), where the content increased statistically significantly (*p* < 0.05). Berasategi et al. [24] also found increased concentrations of myristic fatty acids after heat treatment. The fluctuation (*p* < 0.05) in the content of arachidic (C20:0) and behenic (C22:0) fatty acids was also measured. The overall significant changes in the total content of SFAs were not monitored. Negative significant (*p* < 0.05) correlations between heating cycles and fatty acid contents (extra virgin olive oil heated at 180°C) were measured for the following fatty acids: oleic acid (C18:1), trans oleic acid (C18:1), erucic acid (C22:1), mead acid (C20:3), eicosadienoic acid (C20:2), and docosadienoic acid (C22:2). In contrast, a positive significant (*p* < 0.05) correlation was monitored for the following fatty acids: myristic acid (C14:0), pentadecylic acid (C15:0), stearic acid (C18:0), arachidic acid (C20:0), behenic acid (C22:0), myristoleic acid (C14:1), pentadecenoic acid (C15:1), palmitoleic acid (C16:1), and heptadecenoic acid (C17:1). A statistically significant (*p* < 0.05) decrease occurred in MUFAs and PUFAs after three cycles of heating. It was stated by previous studies that temperatures of deep frying (>180°C) led to changes in fatty acid composition [18]. In contrast, higher concentrations of oleic acid make olive oil more stable during heating, and also olive oil as a representative of cold-pressed plant oils is one of the most stable oils during the storage period [25]. It can also be explained by the fact that olive oil contains lower amounts of PUFAs, such as linoleic (C18:2) and linolenic (C18:3) acids that oxidize rapidly during storage and heating.

**Table 1:** Fatty acid profile of virgin olive oil heated in three cycles at 180°C

	Virgin olive oil 180°C			
	Control	First heating	Second heating	Third heating
	Mean ± SD (mg/mL)	Mean ± SD (mg/mL)	Mean ± SD (mg/mL)	Mean ± SD (mg/mL)
Ʃ SFA	26.62 ± 4.91	24.15 ± 4.89	25.62 ± 7.59	26.08 ± 8.28
Ʃ MUFA	46.01 ± 6.36^a^	42.62 ± 4.19^ab^	40.08 ± 6.94^ab^	32.31 ± 10.98^b^
Ʃ PUFA	3.56 ± 1.36^a^	2.64 ± 1.10^b^	2.86 ± 0.73^b^	2.82 ± 0.89^b^

Different superscript letters (a, b, c) indicate significance (*p* < 0.05) between columns.

**Table 2:** Fatty acid profile of virgin olive oil heated in three cycles at 220°C

	Virgin olive oil 220°C			
	Control	First heating	Second heating	Third heating
	Mean ± SD (mg/mL)	Mean ± SD (mg/mL)	Mean ± SD (mg/mL)	Mean ± SD (mg/mL)
Ʃ SFA	26.68 ± 4.91	22.82 ± 6.26	22.22 ± 8.09	22.06 ± 6.70
Ʃ MUFA	46.01 ± 6.36^a^	19.14 ± 9.00^b^	18.63 ± 6.90^b^	18.72 ± 7.38^b^
Ʃ PUFA	3.56 ± 1.36^a^	3.27 ± 1.71^ab^	2.74 ± 0.62^b^	2.97 ± 0.47^b^

Different superscript letters (a, b, c) indicate significance (*p* < 0.05) between columns.

**Table 3:** Fatty acid profile of refined olive oil heated in three cycles at 180°C

	Refined olive oil 180°C			
	Control	First heating	Second heating	Third heating
	Mean ± SD (mg/mL)	Mean ± SD (mg/mL)	Mean ± SD (mg/mL)	Mean ± SD (mg/mL)
Ʃ SFA	25.82 ± 1.90	24.76 ± 3.86	24.12 ± 4.44	31.32 ± 5.06
Ʃ MUFA	47.18 ± 3.78	43.04 ± 4.26	41.50 ± 7.50	40.54 ± 4.87
Ʃ PUFA	5.34 ± 0.94^a^	3.23 ± 1.62^a^	2.41 ± 0.60^a^	2.18 ± 0.38^b^

Different superscript letters (a, b, c) indicate significance (*p* < 0.05) between columns.

**Table 4:** Fatty acid profile of refined olive oil heated in three cycles at 220°C

	Refined olive oil 220°C			
	Control	First heating	Second heating	Third heating
	Mean ± SD (mg/mL)	Mean ± SD (mg/mL)	Mean ± SD (mg/mL)	Mean ± SD (mg/mL)
Ʃ SFA	25.81 ± 1.90	22.42 ± 4.98	21.21 ± 7.86	19.20 ± 4.83
Ʃ MUFA	47.18 ± 3.78^b^	36.87 ± 5.47^a^	16.44 ± 7.30^a^	17.34 ± 4.90^a^
Ʃ PUFA	5.34 ± 0.94^a^	2.58 ± 0.44^ab^	2.38 ± 1.22^b^	2.36 ± 0.21^b^

Different superscript letters (a, b, c) indicate significance (*p* < 0.05) between columns.

That is the reason why olive oil is often marked as an oil with properties of better resistance toward thermal treatment. Certainly, heating period and heating time affect differently the fatty acid profiles of cooking oils, since heating for 5 min is considered short term and heating for 40 min and more is considered long term. Previous studies indicated that especially these long-term heating resulted in increments of SFA contents [25,26]. In contrast, other studies are also indicating that the fatty acid profile is more affected by cooking temperatures than the heating time [16,27]. On the other hand, the study of Allouche et al. [28] indicated that olive oil samples heated at 180°C for 6 h demonstrated changes in palmitoleic, linoleic, and linolenic acids, but changes in oleic acid contents were not monitored.

It can be observed from Table 2 that heat treatment at 220°C resulted in more significant (*p* < 0.05) decrease in MUFAs and PUFAs in extra virgin olive oils. The difference between heat treatments (180°C and 220°C) is clearly visible since the second cycle of heat treatment at 220°C resulted in a content of omega-3 fatty acid (C18:3 – linolenic fatty acid) under the detectable limit. In contrast, the content of lauric fatty acid (C12:0) stayed almost intact during the three heating cycles at both 180°C and 220°C in the samples of extra virgin olive oil. Lauric fatty acid is a medium chain fatty acid and studies have showed that it has a strong antimicrobial activity [29].

Myristic acid (C14:0) content increased significantly (*p* < 0.05) during heating cycles, and it also showed statistically significant (*p* < 0.01) positive correlations (*r* = 0.374) among three heating cycles. During heating cycles, the same trends were found for pentadecylic acid (C15:0), stearic acid (C18:0), behenic acid (C22:0), and for arachidic acid (C20:0), with significant (*p* < 0.01) positive correlation factor (*r* = 0.658), which is similar to tricosylic acid (C23:0) and lignoceric (C24:0) acid.

The content of palmitic acid (C16:0) also demonstrated statistically significant decrease (*p* < 0.05), with a negative correlation (*p* < 0.01) factor of −0.512. Statistically significant (*p* < 0.01) negative correlation (*r* = −0.850) was calculated from decreased amounts of oleic acid (C18:1) during three heating cycles. After three repeated heating cycles, the following PUFAs were under the detection limit: linolenic acid (C18:3), arachidonic acid (C20:4), and eicosapentaenoic acid (C20:5). Statistical (*p* < 0.05) decreases and negative correlations (*p* < 0.05) were also found during the three heating cycles for eicosadienoic acid (C20:2) and docosadienoic acid (C22:2; Table 2). These changes are at the lower rate among the samples of extra virgin olive oil samples heated during three cycles at 180°C (Table 1). Heat treatments significantly affect the fatty acid profiles of olive oil, which is similar to other plant oils. Plant oils containing higher contents of MUFAs, especially oleic acid (C18:1) are considered more heat stable. Extra virgin olive oil with its higher content of polyphenolic compounds is also, according to studies, considered more stable during heating [9,10,22]. On the other hand, higher concentrations of SFAs in oils make them more resistant toward oxidation. The health benefits of MUFAs are also reflected in the findings, that is, their consumption reduced the level of bad cholesterol in blood, and therefore, PUFAs are essential for the proper functioning of the human organs [30].

Palmitic acid (C16:0) content increased only in the samples of refined olive oil after three cycles of heating at 180°C (Table 3). In the study of Giuffrè et al. [30], an increase in palmitic acid was also observed (C16:0), at 240°C, for 120 min. The total content of SFAs in the same study [30] also increased at the same temperature regime, while in our study SFAs increased only in the samples of refined olive oil after three heating cycles at 180°C, and this was not statistically significant (*p* > 0.05; Table 3).

Linoleic acid (C18:2) showed a decreased trend in almost all investigated samples (Tables 1, 3, and 4), which is similar to the findings of Giuffrè et al. [30]. The samples of extra virgin olive oil that passed through three heating cycles at 220°C had a slightly increased content of linoleic acid (C18:2), which was not statistically significant (*p* > 0.05). Positive correlations were observed mainly among SFAs, especially stearic acid (C18:0; *r* = 0.655; *p* < 0.05; Table 3).

The content of oleic fatty acid decreased statistically significantly (*p* < 0.05) with significant (*p* < 0.01) negative correlation (*r* = −0.675).

In the refined olive oil samples, PUFA is lost after three heating cycles at 180°C and this occurred at a higher rate (*p* < 0.05) compared to that in extra virgin olive oil samples; while the three heating cycles at 220°C also resulted in significant (*p* < 0.01) decrease in MUFA. Heat processing reflects changes in the fatty acid profile of plant oils, and the same holds good for cold pressed and refined oils. It also reflects the sensory properties of oils due to hydrolysis, oxidation, and polymerization. These processes affect the oil unfavorably, especially the extra virgin olive oil [18]. The differences between the extra virgin olive oil and the refined olive oil can be observed based on their antioxidant profiles. Various studies differently declare the role of antioxidants in extra virgin olive oil during its heat treatment.

Certain studies are also stating that compared to refined plant oils, cold-pressed plant oils are less stable during heating [30,31]. The heating at 220°C during three heating cycles resulted in a decrease (*p* < 0.05) in amount of palmitic acid (C16:0) with negative significant (*p* < 0.01) correlation (*r* = −0.821). The same trend was observed with the amounts of oleic acid (C18:1) and linoleic acid (C18:2) that decreased (*p* < 0.05) with negative correlations (*p* < 0.01; *r* = −0.957 and *r* = −0.725, respectively; Table 4). Fatty acids represent a very important indicator of nutritional profile and quality of each oil. This profile is influenced differently according to the type of storage and especially based on the processing types that include heat treatments. Combinations of temperatures and application time affect differently the fatty acid profile changes [32,33]. Linoleic (C18:2) and linolenic (C18:3) fatty acids belong to fatty acids that degrade more rapidly during heating processes. Certainly, the results can vary based on the different olive cultivars [25].

The results gained from the experiment are in accordance with Gharby et al. [25] since the authors also found more changes in MUFA and PUFA in the samples of refined olive oil compared to the extra virgin olive oil. It can be explained by the fact that extra virgin olive oil contains higher amounts of antioxidant compounds (chlorophylls, carotenoids, and especially polyphenols) that protect unsaturated fatty acids, especially PUFA from deterioration, though the processes around polyphenol behaviors during heating in different food matrixes are still not fully understood [25,34].

**Figure 2:**
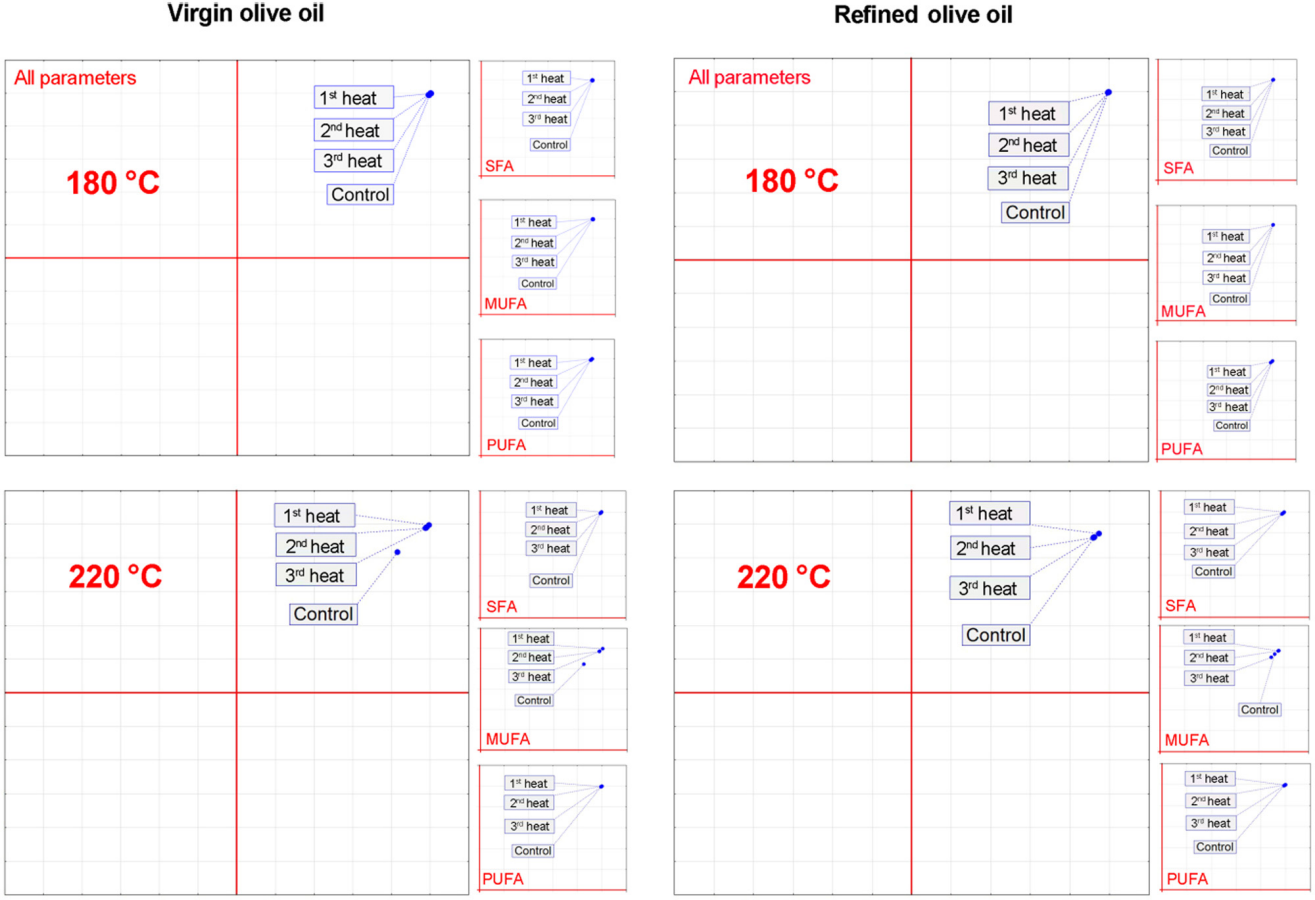
PCA of fatty acid profiles changes during heating treatment of extra virgin and refined olive oils.

The results obtained by PCA are shown in Figure 2. The PCA results indicate that the biggest changes in fatty acid profiles were observed among extra virgin olive oil samples heated at 220°C, far more than the differences observed between refined olive oil samples undergoing three repeated heating cycles at 220°C (Figure 2).

**Figure 3:**
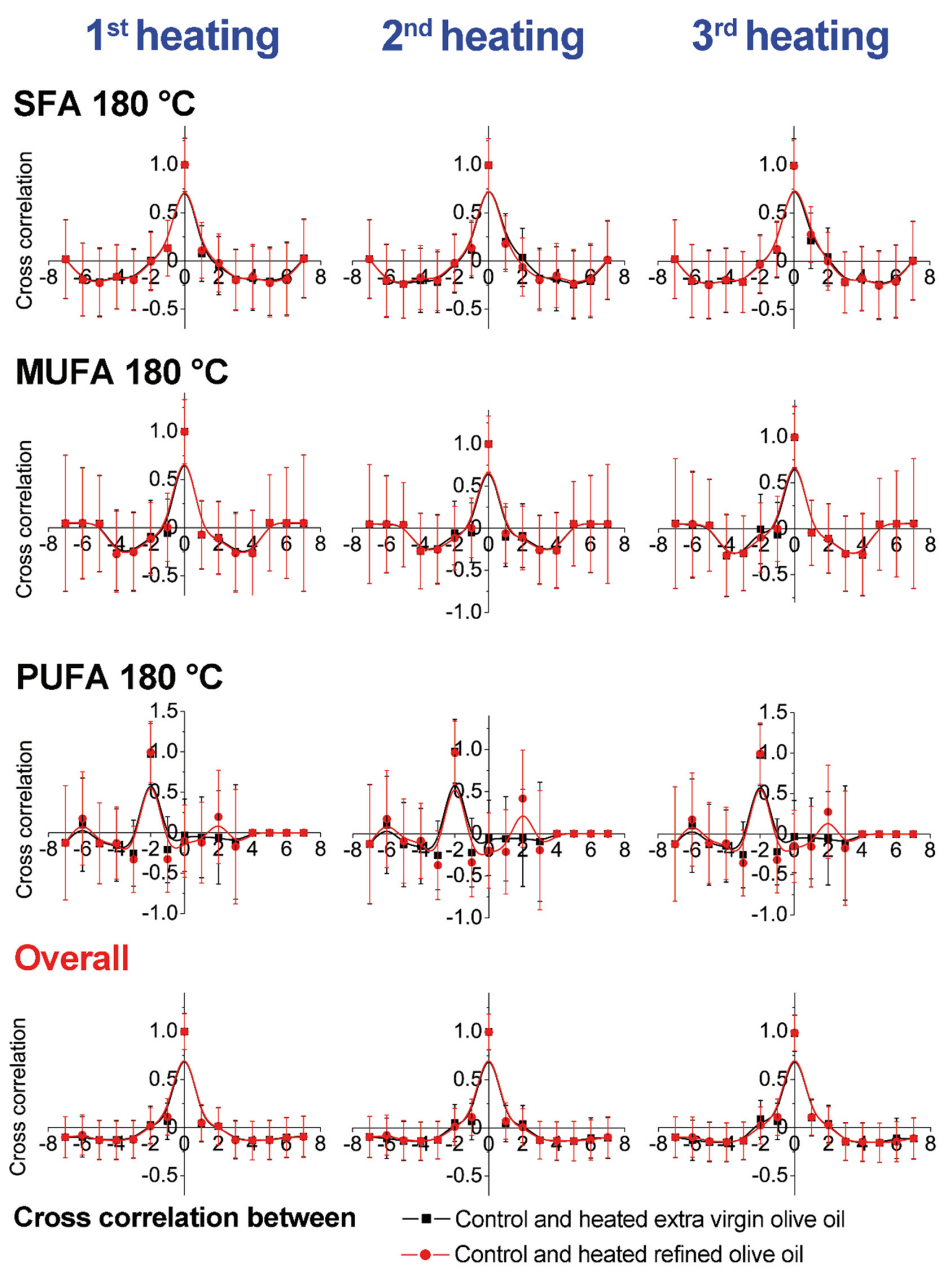
Cross-correlation analysis of olive oil samples within same groups (extra virgin and refined olive oils) heated during three repeated cycles at 180°C.

**Figure 4:**
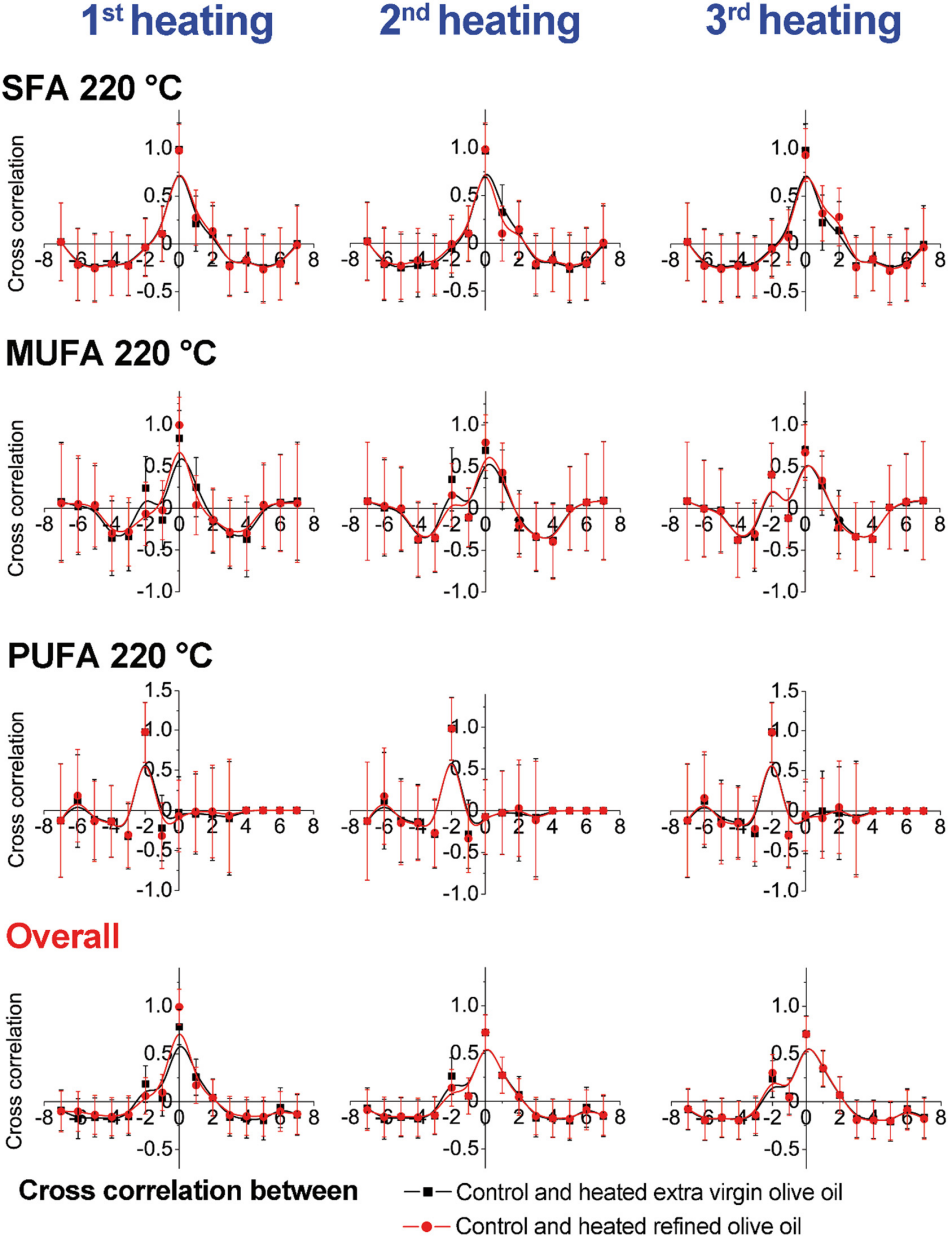
Cross-correlation analysis of olive oil samples within same groups (extra virgin and refined olive oils) heated during three repeated cycles at 220°C.

**Figure 5:**
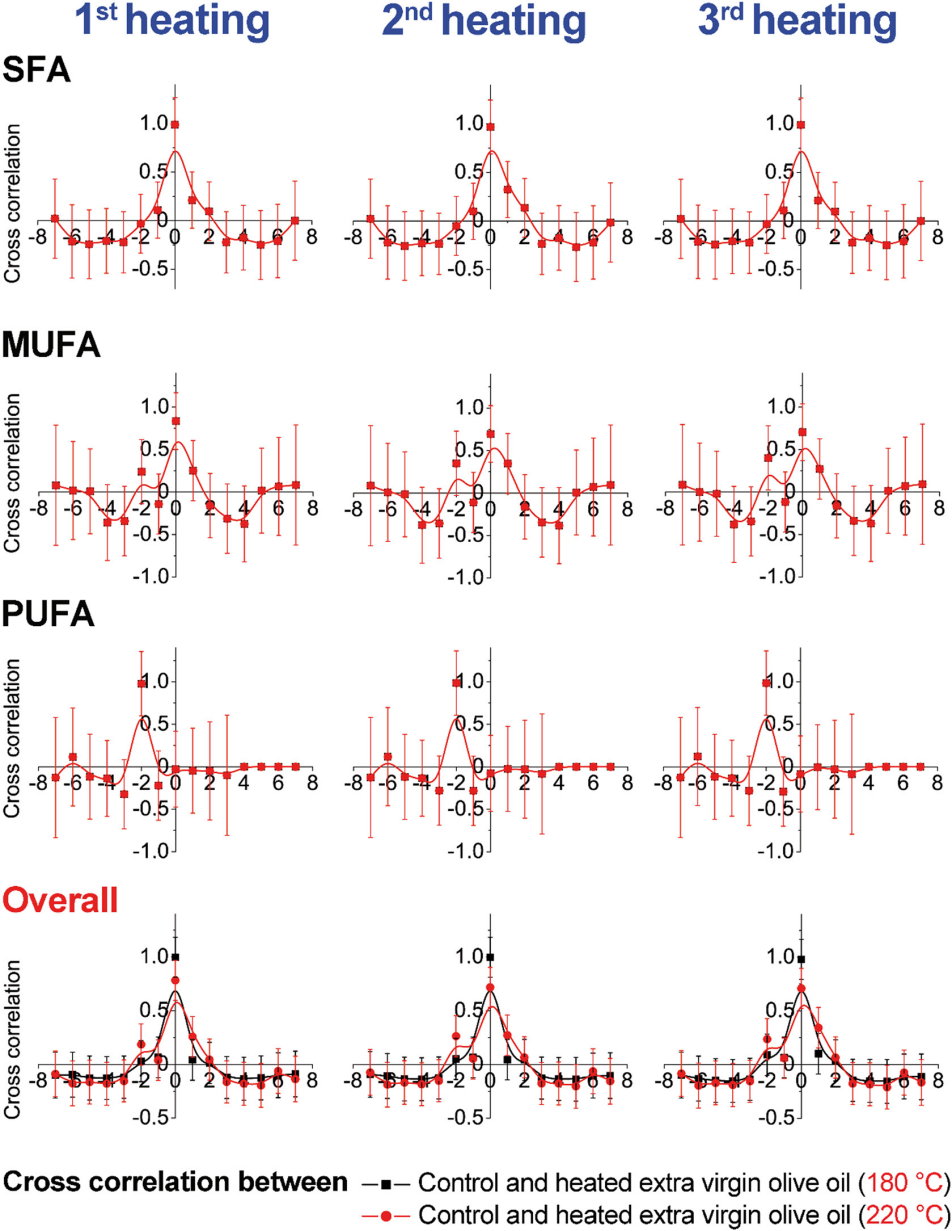
Cross-correlation analysis of olive oil samples between groups (extra virgin olive oils) heated during three repeated cycles at 180°C and 220°C.

**Figure 6:**
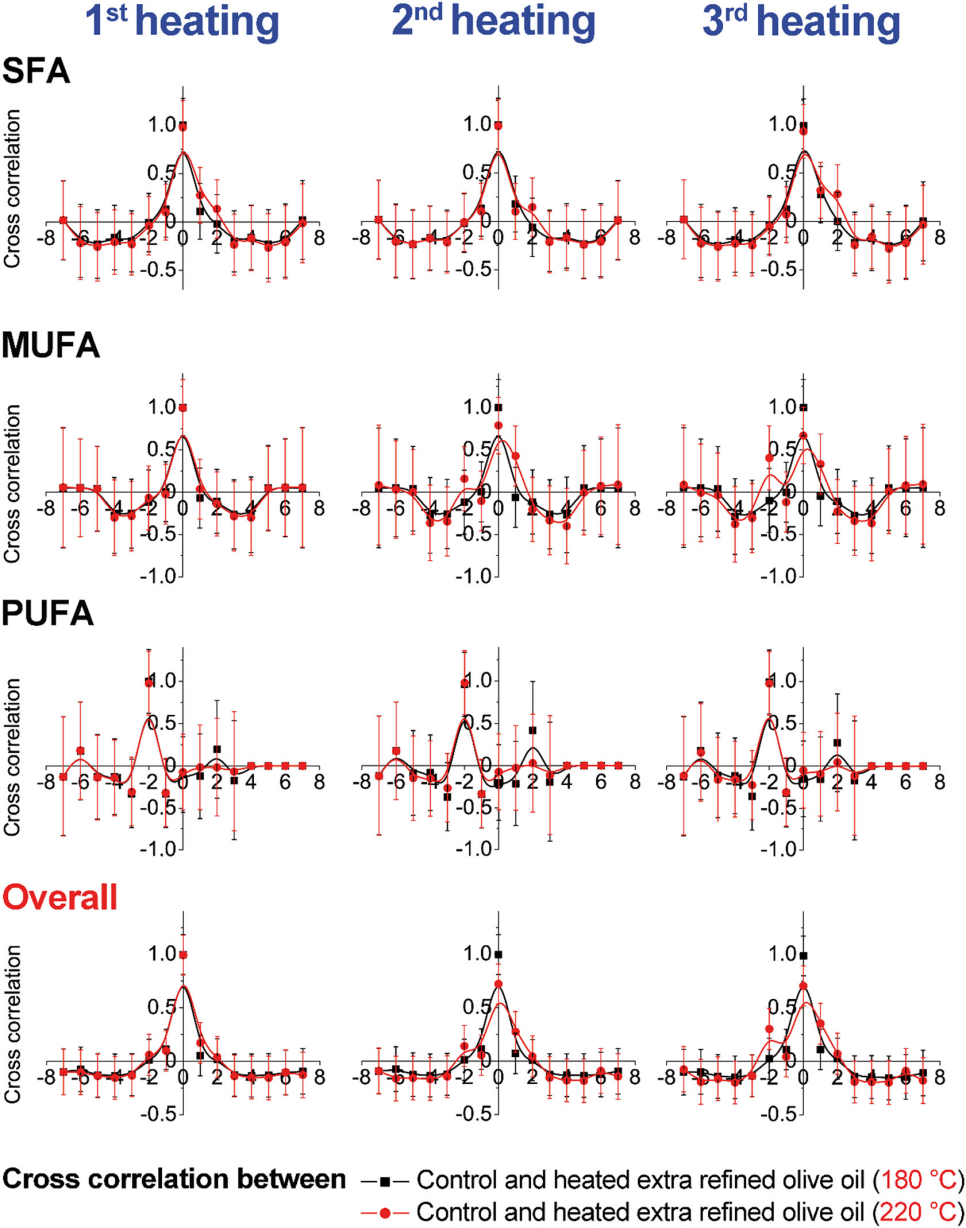
Cross-correlation analysis of olive oil samples between groups (extra refined olive oils) heated during three repeated cycles at 180°C and 220°C.

Cross-correlation results presented graphically are shown in Figures 3–6. The obtained results emphasize changes within the same group and between two examined olive oil groups (extra virgin and refined olive oils). The results indicate higher changes in MUFAs at 220°C in comparison with the results of samples heated at 180°C, within the refined olive oil samples. In contrast, cross-correlation results of extra virgin olive oil heated at 180°C and 220°C indicated higher changes among PUFAs (Figures 3 and 4). According to cross-correlation results, higher differences were observed in the content of MUFAs and PUFAs between refined olive oil samples heated at 180°C and 220°C. The overall differences were not observed between the examined two types of olive oil (Figures 5 and 6).

**Figure 7:**
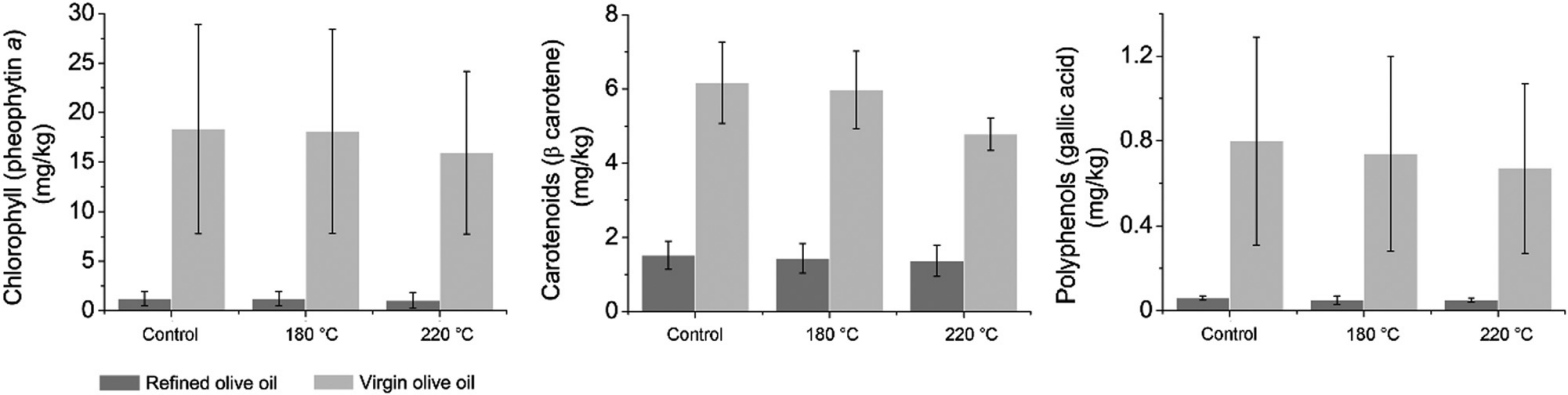
Chlorophyll, carotenoid, and polyphenolic compound contents in olive oil before and after three cycles of heating at 180°C and 220°C.

**Figure 8:**
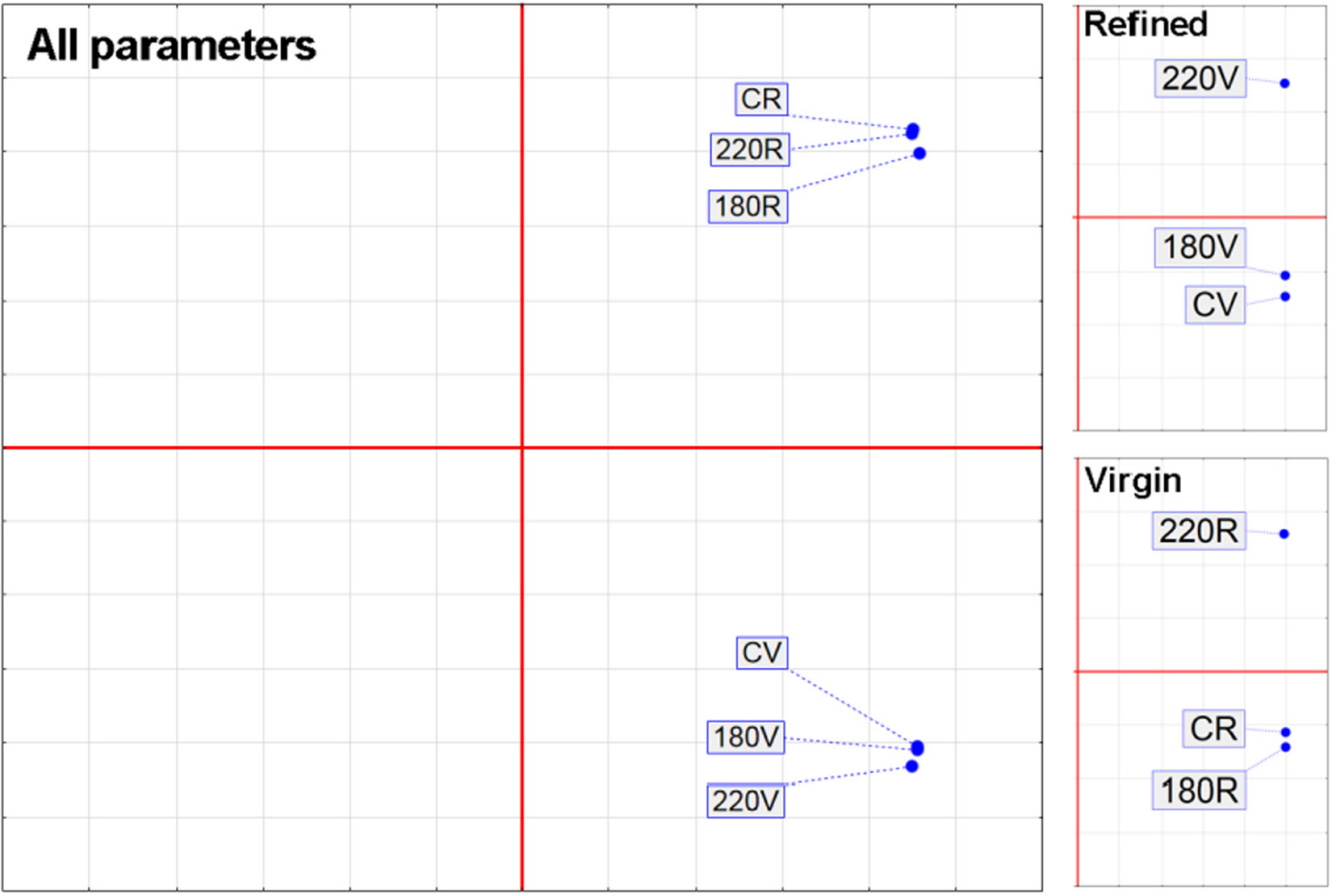
PCA of antioxidant contents in olive oil before and after three cycles of heating at 180°C and 220°C. CR – control samples; 180R – refined oils heated at 180°C; 220R – refined oils heated at 220°C; 180V – virgin oils heated at 180°C; 220V – virgin oils heated at 220°C.

Antioxidant profile changes in olive oil samples (both refined and virgin olive oils) before and after three cycles of heating are shown in Figures 7 and 8. The trend of reducing chlorophyll, carotenoids, and polyphenols contents can be clearly seen in Figure 7. The differences between the three heating cycles can also be observed between olive oil samples heated at 180°C and 220°C (Figure 8).

PCA formed separate groups of olive oil samples heated at 220°C. The olive oil, especially cold-pressed olive oil, has a unique flavor due to the polyphenol content that positively affects the oxidative stability of olive oil [25]. It is known that polyphenols are the most affected due to heat treatment, though the reductions differ based on the olive cultivar [28].

## 4 Conclusions

The experiments showed that the cycles of heating temperatures at 180°C and at 220°C affect the fatty acid profile of olive oils. Certainly, our results unambiguously emphasized how relatively small differences in heating temperatures result in totally different fatty acid profiles. The study also indicated that extra virgin olive oil can be used for culinary heating in the same way as refined olive oil, though only at a lower temperature below 180°C. Refined olive oil also did not show high stability during heating at 220°C. Cross-correlation analysis emphasized the differences between extra virgin and refined olive oil during heating, i.e., both between them and within the same olive oil group. MUFAs, according to cross-correlation analysis, changed more rapidly in refined olive oil, while PUFAs changed more rapidly in extra virgin olive oil. The changes in antioxidant compounds (chlorophyll, carotenoids, and polyphenols) after and before the three heating cycles also showed statistical reduction, especially among samples heated at 220°C (in both refined and virgin olive samples).
